# Fermented marigold meal enhances muscle nutrition and flavor in white feathered broilers via gut microbiota modulation

**DOI:** 10.1016/j.fochx.2025.103165

**Published:** 2025-10-14

**Authors:** Zezhu Du, Yan Shen, Jinya Dong, Siyu Zhou, Yuanfeng Chen, Huiqing Luo, Shikuan Zhao, Zhiyu Li, Cheng Gong, Lihui Yu, Xiaocui Du, Tianjun Li, Yunfei Ge, Ruijuan Yang, Chongye Fang

**Affiliations:** a College of Food Science and Technology, Yunnan Agricultural University, Kunming 650201, China; b Yunnan Research Center for Advanced Tea Processing, Yunnan Agricultural University, Kunming 650201, China; c College of Agronomy and Biotechnology, Yunnan Agricultural University, Kunming 650201, China

**Keywords:** Marigold, White feather broilers, Meat quality, Flavor compounds, Fatty acid, Gut microbiota

## Abstract

Marigold (*Tagetes erecta* L.) residue, a by-product of industrial lutein extraction, is rich in carotenoids and polyphenols, offering potential as a poultry feed additive. This study employed *Lactobacillus plantarum* for solid-state fermentation of marigold meal and evaluated dietary inclusion (5 % or 10 %) in broilers for 42 days. Fermented marigold meal had no significant effects on growth performance or basic meat quality (pH, color, water-holding capacity) but improved breast muscle composition by increasing non-essential amino acids (e.g., glutamate, GABA), n-3 PUFAs, and PUFA/SFA ratio, while reducing MUFAs. 16S rRNA sequencing revealed enrichment of beneficial bacteria (*Parabacteroides*, *Lactobacillus*) and higher butyric acid levels. GC–MS identified 171 volatile compounds, with elevated key aroma compounds (e.g., 2(3H)-furanone, Geranylacetone) in marigold-fed groups, linked to altered microbiota. Fermented marigold meal enhances meat nutrition and flavor through gut microbiota-mediated pathways, providing a sustainable approach for high-value utilization of agricultural by-products.

## Highlights

• First evaluation of fermented *Tagetes erecta* residue for animal feed application.
• Fermentation enriched total flavonoids, phenolics, sugars, and protein in marigold residue.
• Dietary fermented residue improved broiler muscle GABA and n-3 PUFA content.
• Gut microbiota was reshaped, increasing *Lactobacillus* and SCFA (butyrate) levels.
• A “microbiota–metabolism–muscle deposition” mechanism was proposed and verified.

## 1 Introduction

According to the OECD-FAO Agricultural Outlook 2022–2031, the global shift in meat consumption toward poultry is projected to continue strengthening. Global poultry production is anticipated to increase by 16 % by 2031, with poultry accounting for 47 % of total meat protein consumption (Organization for Economic, Development, Food & Agriculture Organization of the United, 2022). China ranks as the world's second-largest chicken producer; however, its export volume is lower than that of Brazil, the United States, and the European Union(Govoni et al., 2021), primarily due to increasing domestic demand. Particularly following the COVID-19 pandemic, poultry meat has been favored for its low-fat and high-protein content, aligning with heightened health consciousness among consumers(Cui et al., 2022; Xu & Yin, 2024). Additionally, chicken is regarded as a healthier alternative to red meat due to its affordability, high nutritional value, favorable sensory attributes, and lower levels of fat, cholesterol, and calories, making it a valuable component of a balanced diet(Goethals et al., 2019). However, the intensive pursuit of accelerated growth rates in broiler chickens to meet market demand has led to the prolonged use and, in some cases, misuse of antibiotics during poultry farming(Roskam, Oude Lansink, & Saatkamp, 2020). Such practices have been prohibited in several countries(Bosetti et al., 2020). In recent years, probiotics, prebiotics, and key phytochemicals—such as polyphenols, polysaccharides, and alkaloids—have demonstrated beneficial effects on poultry growth(Fan et al., 2018). Plant-derived additives can be utilized individually or synergistically in poultry production to modify feed palatability, enhance nutrient absorption, modulate immune responses, improve growth performance and meat quality, and regulate gut microbiota(Kim & Ahn, 2022).

Marigold (*Tagetes erecta* L.), a member of the Asteraceae family, is widely cultivated in countries such as Mexico, China, and Zambia. It is regarded as one of the most significant ornamental plants and industrial crops globally(Panda, Debnath, Mall, Nigam, & Rao, 2021). Studies have indicated that marigold stems contain approximately 26.5 % crude protein, 5 % crude fat, and 35.1 % crude fiber(Hou et al., 2021). Currently, marigold petals serve as the primary industrial source of lutein (Zheng et al., 2022), containing a total lutein content ranging from 88 % to 92 %. However, approximately 40,000 tons of marigold residues are discarded annually as waste or utilized solely as fertilizer, leading to significant environmental pollution and substantial wastage of natural resources(Wu et al., 2023). Reports indicate that marigold residues are rich in bioactive compounds such as caffeic acid, gallic acid, flavonoids, and flavonoid-O-glycosides(Gong et al., 2012). Previous studies have demonstrated that lutein not only enhances skin pigmentation but also possesses potent antioxidant properties, making it beneficial across various species(S. Wang et al., 2017). Despite these nutritional and functional properties, the potential of marigold residues as feed additives for poultry remains underexplored, particularly in relation to meat flavor improvement.

Flavor is a critical determinant of meat quality and consumer acceptance(Liu & Fang, 2022). During cooking, chemical reactions such as the Maillard reaction and lipid oxidation generate volatile organic compounds (VOCs), which are key contributors to meat aroma(Sun et al., 2024; Zhang et al., 2023). Current analytical approaches to VOCs combine sensory evaluation with instrumental techniques, with gas chromatography–mass spectrometry (GC–MS) serving as a powerful tool for separating and identifying complex volatile profiles due to its high resolution and sensitivity in both qualitative and quantitative detection (X. Yang et al., 2024). GC–MS analysis of fried chicken breast has identified compounds such as 2-acetylthiophene, bis(2-methyl-3-furyl)disulfide, and (E,E)-2,4-decadienal as key contributors to fried chicken flavor, while aldehydes (particularly 2,4-dienal and 2-enal), ketones, heterocyclic compounds, and sulfides also play crucial roles(Flores, 2018; Shakoor et al., 2023). High-temperature frying, especially in the presence of cooking oil, further enhances these flavor characteristics (Y. Wang et al., 2024). However, to the best of our knowledge, few studies have in*v*estigated how feeding broilers with marigold meal influences the generation of VOCs during deep-frying of breast muscle. Therefore, this study aimed to characterize the changes in VOCs and lipid profiles in fried chicken breast from marigold-fed broilers, with the goal of elucidating potential mechanisms for controlling lipid oxidation and enhancing meat flavor.

Our study aimed to evaluate the effects of fermented marigold meal supplementation on broiler performance, muscle nutritional composition, volatile flavor compounds of the meat, and cecal microbiota. We further sought to elucidate potential mechanisms linking gut microbiota modulation to improvements in meat quality. We hypothesized that fermented marigold residue, as a functional feed ingredient, would enhance muscle nutrient deposition and favorably alter the flavor profile of chicken meat through its impact on the intestinal microbiome.

## 2 Materials and methods

### 2.1 Preparation of test materials

The marigold raw materials were supplied by Luoping Yihong Biotechnology Co., Ltd. Marigold meal, obtained as the residual by-product following lutein extraction, was used in this study (Supplementary Fig. S1). The preparation process for fermented marigold meal involved mixing the marigold meal with distilled water at a ratio of 2:5 (*w*/*v*), sterilizing the mixture at 120 °C for 15 min, inoculating it with *Lactobacillus plantarum* at 1:15 % (*w*/w), fermenting at 38–42 °C for 72 h, followed by drying for 2–4 days and subsequent pulverization.

### 2.2 Determination of the chemical composition of marigold meal

Total flavonoid, total sugar, and total phenol content were determined by improved methods(Sultana et al., 2025). The total protein content was determined by the BCA microplate method of protein quantification (TP) assay kit. Lutein content is carried out concerning improved methods(Manzoor, Rashid, Prasad Panda, Sharma, & Azhar, 2022).

### 2.3 Characterization of marigold meal structure

The structural characteristics of marigold meal were analyzed using X-ray diffraction (XRD). The instrument (Rigaku Miniflex 600, Japan) was preheated and stabilized for approximately 2 min prior to scanning. Samples were mounted on the sample holder, and diffraction data were collected over a 2θ range of 5–90° at a scanning rate of 5°/min.

Functional groups and chemical components of marigold meal were identified using Fourier-transform infrared (FTIR) spectroscopy. Approximately 10 mg of dried sample was thoroughly mixed with spectroscopic-grade potassium bromide (KBr), ground in an agate mortar, and compressed into transparent pellets using a tablet press. Background correction was performed prior to analysis, and spectra were collected using an FTIR spectrometer (Bruker Tensor 27, Germany) within a spectral range of 4000–400 cm^−1^.

The surface morphology of marigold meal was examined using a high-resolution field emission scanning electron microscope (FE-SEM). Samples were mounted onto conductive adhesive and sputter-coated with gold using a vacuum coater. Imaging and elemental analysis were performed using a FE-SEM (ZEISS Sigma 300, Germany) equipped with an energy-dispersive X-ray spectroscopy (EDS) detector (Oxford Instruments).

### 2.4 Experimental design and feeding management

Ninety male white-feathered broiler chickens (initial body weight: 102.28 ± 1.11 g), provided by Yunnan Fengzhen Agriculture and Animal Husbandry Co., Ltd., were randomly assigned to five experimental groups, with three replicates per group and six birds per replicate. The five dietary treatments were: CK (control group, soybean meal-based diet), MML (soybean meal +5 % marigold meal), MMH (soybean meal +10 % marigold meal), MFL (soybean meal +5 % fermented marigold meal), and MFH (soybean meal +10 % fermented marigold meal). Birds were housed in stainless steel cages under natural ventilation, with ad libitum access to feed and water. The indoor relative humidity was maintained at approximately 50 %. The ambient temperature was initially maintained at 35 °C during the first 7 days and then gradually reduced by 2–3 °C per week until the end of the 42-day feeding trial. Throughout the experimental period, continuous lighting was provided. Daily feed intake was recorded, and body weight was measured weekly. A basal diet (Table S1) was provided during both the starter phase (days 0–21) and the finisher phase (days 22–42). Animal experiments were approved by the Animal Ethics Committee of Yunnan Agricultural University, and Approval No.: APYNAU 202505047. All animal experiments comply with the ARRIVE guidelines and are conducted in accordance with the UK Animals (Scientific Procedures) Act 1986 and associated guidelines, the EU Directive on animal experimentation 2010/63/EU, or the National Research Council's Guidelines for the Care and Use of Laboratory Animals.

### 2.5 Sample collection

Samples were collected at the end of the 42-day feeding trial. Following a 12-h fasting period, the final body weight of each broiler was recorded. These broilers were euthanized through exsanguination using a conventional neck cut. Bleeding was allowed for 3–5 min prior to tissue collection. The left pectoralis major muscle was used to assess meat quality parameters, including pH, color (chroma), shear force, and moisture content, as well as for the determination of amino acid and fatty acid composition. The right pectoralis major muscle was used to analyze nutritional components, specifically crude fat and crude protein contents. Cecal contents were collected into sterile 2 mL cryotubes, snap-frozen in liquid nitrogen, and stored at −80 °C for subsequent microbiota and metabolite analyses.

### 2.6 Indexes

#### 2.6.1 Growth performance

During the experimental period, feed offered and feed residues were recorded daily. Broilers were weighed weekly to monitor growth. Growth performance indicators, including average body weight (BW), average daily gain (ADG), average daily feed intake (ADFI), and feed to weight ratio (F/G), were calculated based on data collected over the 42-day trial period.

$$\mathrm{ADG}\;\left(g\right)=\left(\mathrm{final weight}-\mathrm{initial weight}\right)/\mathrm{test days}$$

$$\mathrm{ADFI}\;\left(g\right)=\left(\mathrm{total feed intake}\right)/\mathrm{test days}$$

$$F/G\;\left(g\right)=\mathrm{ADFI}/\mathrm{ADG}$$

#### 2.6.2 pH value measurement

The left pectoralis major muscle from six broilers per treatment group was collected and measured using a portable waterproof pH meter (HI9124; Hanna Instruments, Inc., China) with a resolution of 0.01. The instrument was calibrated prior to use with standard buffer solutions at pH 4.01, 6.86, and 7.01.

The pH electrode was inserted into three distinct locations in the left pectoralis major muscle at two time points: 45 min and 24 h postmortem. Each sample was measured in triplicate at each time point, and the average value was used for further analysis.

#### 2.6.3 Determination of flesh color

The left pectoralis major muscle was sampled from six broilers per treatment group. Color parameters—luminance (L*), redness (a*), and yellowness (b*)—were measured at 45 min and 24 h postmortem using a portable colorimeter (CR-410; Konica Minolta, Japan). Each sample was measured in triplicate at each time point, and the mean value was used for statistical analysis.

#### 2.6.4 Determination of cooking loss

Left pectoralis major muscle samples of uniform size were collected from each of the six treatment groups. The samples were gently blotted dry and weighed to obtain the initial mass (m1), then wrapped in aluminum foil and cooked in a water bath at 80 °C for 15 min. After cooling to room temperature (25–30 °C), surface moisture was removed using absorbent paper, and the cooked samples were weighed (m2). Cooking loss (%) of the pectoralis major muscle was calculated using the following formula:

$$\mathrm{Cooking loss}\;\left(\%\right)=\left(m1-m2\right)/m1\times 100\%$$

#### 2.6.5 Measurement of drip loss

Left pectoralis major muscle samples were collected from each of the six treatment groups and cut into uniform blocks (3 × 2 × 1 cm). Surface connective and adipose tissues were carefully trimmed, and surface moisture was removed using absorbent paper. The initial weight of each sample was recorded as m3. Each sample was suspended using a paper clip in a 4 °C refrigerator and loosely wrapped in plastic film to avoid direct surface contact. After 24 h, surface exudate was gently remo*v*ed with absorbent paper, and the final weight was recorded as m4. Drip loss (%) was calculated using the following formula:

$$\mathrm{Drip loss}\;\left(\%\right)=\left(m3-m4\right)/m3\times 100\%$$

#### 2.6.6 Shear force measurement

After cooking loss determination, each pectoralis major muscle sample was cut into three strips with a cross-sectional area of 1 × 1 cm and a length of 5 cm, parallel to the direction of the muscle fibers. Shear force was measured by cutting perpendicular to the muscle fibers using a digital texture analyzer. Each strip was measured in triplicate, and the mean value was recorded for subsequent analysis.

#### 2.6.7 Determination of chemical composition

(1) The moisture content is determined by the direct drying method concerning (GB 5009.3–2016 National Food Safety Standard-Determination of moisture in food).

(2) Crude protein reference (GB5009.5–2016 national food safety standard-Determination of protein in foods) is determined by the Kjeldahl nitrogen determination method.

(3) Crude fat reference (GB5009.6–2016 national food safety standard-Determination of fat in food) is determined by the Soxhlet extraction method.

### 2.7 Determination of amino acids and fatty acids

Amino acid composition of the pectoralis major muscle was determined using ultra-performance liquid chromatography coupled with tandem mass spectrometry (UPLC-MS/MS). Approximately 50 mg of homogenized muscle tissue was extracted with a precooled acidified methanol–water solution (1:1, *v*/v) containing an isotopically labeled internal standard (Trp-d₃). After vortexing, homogenization, and centrifugation (12,000 ×*g*, 4 °C, 5 min), the supernatant was filtered through a 0.22 μm membrane and analyzed. Chromatographic separation was performed using a Waters BEH C18 column (2.1 × 100 mm, 1.7 μm) under gradient elution. The mobile phases consisted of formic acid in methanol-water mixtures. Detection was carried out in positive electrospray ionization mode using multiple reaction monitoring (MRM)(Liyanaarachchi, Mahanama, Somasiri, & Punyasiri, 2018).

Fatty acids in the pectoralis major muscle were analyzed following lipid extraction and methyl esterification. Briefly, approximately 50 mg of tissue was extracted using a chloroform–methanol solution (2:1, v/v) and homogenized with glass beads. After centrifugation (4 °C), the supernatant was subjected to methylation using sulfuric acid–methanol at 80 °C for 30 min. Following cooling, the fatty acid methyl esters (FAMEs) were extracted with hexane, washed with ice water, dehydrated, and mixed with methyl salicylate as an internal standard. Analysis was conducted on a Thermo Trace 1300-TSQ 9000 GC–MS system equipped with a TG-FAME column (50 m × 0.25 mm × 0.20 μm) under a programmed temperature gradient. Detection was performed in selected ion monitoring (SIM) mode using electron impact (EI) ionization (70 eV). The method followed the protocol described by Hoving et al.(Hoving, Heijink, van Harmelen, van Dijk, & Giera, 2018), with emphasis on temperature control, accurate phase transfer, and methylation efficiency.

### 2.8 Determination of VOCs

Chicken breast samples were heated at 180 °C for 3 min prior to analysis. VOCs were extracted by headspace solid-phase microextraction using a DVB/Carboxen/PDMS (80 μm) fiber on a CTC TriPlus autosampler. Each sample was placed in a 20 mL headspace vial, sealed and incubated at 90 °C. The fiber was exposed to the headspace with agitation at 250 rpm for 20 min and then desorbed in the gas chromatograph injector for 3 min; the GC cycle time was 41 min. Chromatographic separation and detection were performed on a Trace 1610 gas chromatograph coupled to a TSQ 9610 mass spectrometer (Thermo Fisher Scientific, Germany). High-purity helium (>99.999 %) served as the carrier gas at a constant flow rate of 1.0 mL min^−1^. The oven temperature was programmed from 50 °C to 100 °C at 5 °C min^−1^, increased to 150 °C at 3 °C min^−1^ and finally to 240 °C at 10 °C min^−1^ with a 2 min hold. The injector was maintained at 240 °C, and the ion source and transfer line temperatures were 240 °C and 280 °C, respectively. Mass spectra were acquired in electron-ionization mode at 70 eV over an *m*/*z* range of 40–400. Raw data were processed with Deconvolution Plugin software for peak extraction, deconvolution and alignment, and volatile compounds were identified by matching mass spectra and retention indices against the Wiley and NIST 2020 libraries.

### 2.9 DNA isolation and 16S rRNA gene sequencing

Total genomic DNA was extracted from cecal contents using a commercial kit following the manufacturer's protocol. The V3–V4 hypervariable regions of the 16S rRNA gene were amplified by PCR using specific primers and Phusion® High-Fidelity PCR Master Mix. The reaction conditions included an initial denaturation at 98 °C for 1 min, followed by 30 cycles of 98 °C for 10 s, 50 °C for 30 s, and 72 °C for 30 s, with a final extension at 72 °C for 5 min. PCR products were confirmed by 2 % agarose gel electrophoresis, and target fragments were purified. Sequencing libraries were constructed and quantified using a Qubit Fluorometer and qPCR, then sequenced on the Illumina NovaSeq platform (paired-end 250 bp reads) by Novogene (Beijing, China). Raw sequencing data were demultiplexed and quality-filtered using Cutadapt and FASTP (v0.23.1), and merged with FLASH (v1.2.11) to obtain Raw Tags. Chimera sequences were removed by comparing to the SILVA database (for 16S rRNA), resulting in high-quality Effective Tags. Amplicon Sequence Variants (ASVs) were generated using the DADA2 algorithm in QIIME2, followed by taxonomic classification based on reference databases. Species annotation and downstream diversity analyses were conducted within the QIIME2 framework.

### 2.10 Determination of short chain fatty acids

The samples were resuspended with liquid nitrogen and then homogenized with methanol (80 %) and centrifuged at 12,000 rpm for 10 min to remove the protein. The supernatant was added to derivatization reagent (150 μL) and derivatized at 40 °C for 40 min. The sample was diluted by methanol (80 %). Then supernatant (95 μL) was homogenized with 5 μL mixed internal standard solution. Finally, injected into the LC-MS/MS system for analysis.

### 2.11 Data processing and analysis

Data were initially organized in Microsoft Excel and analyzed using SPSS 27.0. One-way analysis of variance (ANOVA) followed by Tukey's multiple comparison test was used to assess statistical significance. Data are presented as Mean ± SEM. GraphPad Prism 9.5.0 was used for graphical representation, and figures were additionally prepared using Origin 2022. Statistical significance was defined as *P* < 0.05; *P* > 0.05 was considered not significant.

## 3 Results

### 3.1 Chemical composition

Table S2 presents the chemical composition of unfermented marigold meal (MM) and fermented marigold meal (MF). The contents of total flavonoid, total sugars, total phenol, and total protein were significantly higher in the MF group than in the MM group (*P* < 0.05), whereas no significant difference was observed in lutein content between the two groups (*P* > 0.05). These findings indicate that fermentation with *Lactobacillus plantarum* can significantly enhance the nutritional and bioactive compound profile of marigold meal, except for lutein content.

### 3.2 Structural characteristics

**Fig. 1:**
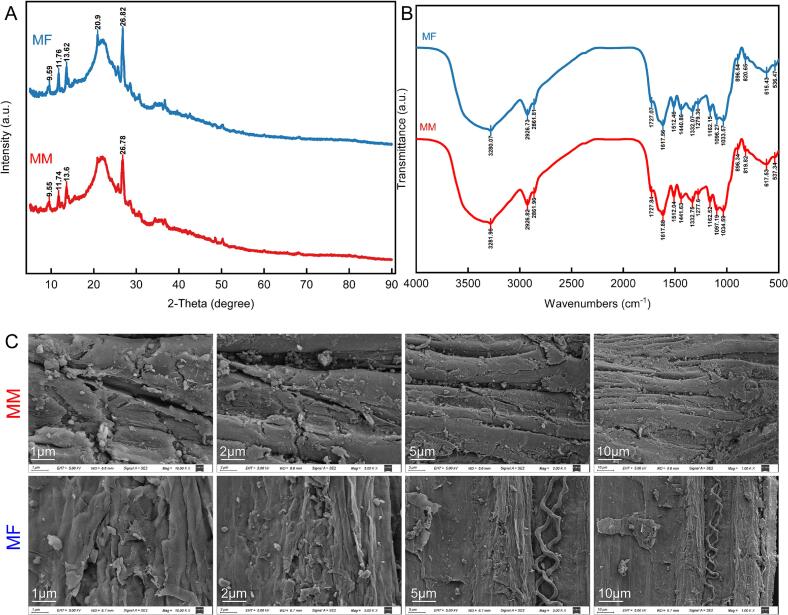
Structural characterization of marigold meal. (A) XRD (B) FTIR (C) SEM

Fig. 1A displays the XRD patterns of MM and MF marigold meal, revealing their crystalline structure characteristics. Within the 2θ scanning range, both samples exhibit multiple diffraction peaks corresponding to different crystal planes. Characteristic peaks were observed at 9.55°, 11.74°, 13.60°, and 26.78° in MM, while MF displayed peaks at 9.59°, 11.76°, 13.62°, 20.90°, and 26.82°, indicating structural alterations induced by fermentation. Fig. 1B shows the FT-IR spectra of MM and MF, reflecting their molecular bond vibrations. Both samples exhibited absorption bands at different wavenumbers. Notably, the broad peak around 3280 cm^−1^ corresponds to O—H or N—H stretching vibrations, and peaks near 2926 cm^−1^ and 2861 cm^−1^ are attributed to C—H stretching. Fig. 1C presents SEM images of MM and MF at various magnifications. The surface of MM appeared rough, with irregular morphology, visible cracks, and fragmented blocks. In contrast, MF exhibited a more compact and homogeneous surface, with relatively smoother texture and altered crack and particle distribution, suggesting that fermentation modified the microstructure of marigold meal.

### 3.3 Growth performance

Table S3 presents the effects of marigold meal supplementation on the growth performance of white feather broilers. The MMH group exhibited the highest ADFI (141.86 g) and F/G (3.81), both of which were significantly higher than those in the CK group (*P* < 0.05). However, no significant differences were observed among groups in initial body weight, final body weight, or ADG (*P* > 0.05). These findings indicate that although marigold meal increased feed intake, it did not enhance weight gain, and thus had no detrimental effect on the overall growth performance of broilers.

### 3.4 Meat quality

Table S4 summarizes the effects of marigold meal supplementation on the breast muscle quality of broilers. The MFH group exhibited the highest drip loss (42.12 %), which was significantly greater than that of the MML group (26.42 %) (*P* < 0.05). Additionally, the MMH group showed a significantly higher cooking loss (31.57 %) compared to the CK group (22.82 %) (*P* < 0.05). However, no significant differences were observed among all groups (CK, MML, MMH, MFL, and MFH) in shear force, pH at 45 min and 24 h postmortem, meat color parameters (L*, a*, b*), crude protein, or crude fat contents (*P* > 0.05).

### 3.5 Muscle amino acids profile

Table 1 displays the amino acid profiles of pectoralis major muscle in broilers. Compared with the CK group, all marigold meal treatment groups showed a significant increase in flavor amino acids (FAA) and total non-essential amino acids (Total NEAA), along with a significant decrease in total essential amino acids (Total EAA) (*P* < 0.05). Notably, lysine (Lys) content decreased in a dose-dependent manner, with the lowest level observed in the MFH group. The concentrations of glutamine (Gln) and glutamic acid (Glu) were significantly elevated in all treatment groups relative to CK, with fermented marigold meal showing a stronger enhancing effect. Remarkably, the MFH group exhibited the highest γ-aminobutyric acid (GABA) content (*P* < 0.001). Proline (Pro) content showed a U-shaped trend: it reached its lowest level in the MFL group but partially recovered in the MFH group.

**Table 1:** Effect of different dosage marigold meal on amino acids in breast muscle of white feather broilers (%).

Items	CK	MML	MMH	MFL	MFH	SEM	*P*-value
EAA							
Met	1.46^a^	1.23^b^	1.04^b^	1.05^b^	1.14^b^	0.04	0.001
Val	2.67^a^	2.20^b^	2.09^b^	1.96^b^	2.25^b^	0.07	0.001
Lys	1.17^a^	0.90^b^	0.79^c^	0.76^c^	0.54^d^	0.05	<0.001
Ile	1.78^a^	1.44^b^	1.29^b^	1.24^b^	1.31^b^	0.05	<0.001
Phe	2.77^a^	2.23^b^	2.07^b^	2.01^b^	2.24^b^	0.07	<0.001
Leu	3.18^a^	2.52^b^	2.29^b^	2.16^b^	2.47^b^	0.10	<0.001
Trp	0.75^a^	0.64^b^	0.60^b^	0.61^b^	0.53^c^	0.02	<0.001
Thr	1.43^a^	0.83^bc^	1.00^b^	0.82^c^	0.90^bc^	0.06	<0.001
His	13.16	12.47	12.97	13.63	12.99	0.22	0.685
NEAA							
Gln	23.44^b^	30.93^a^	31.92^a^	30.98^a^	31.45^a^	1.03	0.016
Gly	3.58^a^	2.44^b^	2.20^b^	2.43^b^	2.29^b^	0.14	<0.001
Pro	1.64^a^	1.28^b^	1.34^b^	1.04^c^	1.63^a^	0.06	<0.001
Arg	1.21^a^	0.98^b^	0.84^cd^	0.88^bc^	0.75^d^	0.04	<0.001
Glu	2.50^d^	2.98^c^	3.08^bc^	3.35^a^	3.22^ab^	0.08	<0.001
Ser	6.05^a^	4.53^b^	4.96^b^	4.55^b^	4.30^b^	0.18	0.003
Tyr	7.89^a^	7.33^ab^	7.18^ab^	6.43^b^	7.78^a^	0.16	0.009
Ala	21.70	21.56	20.96	22.56	20.06	0.29	0.052
Asp	2.16^ab^	2.03^b^	1.87^b^	2.08^ab^	2.62^a^	0.08	0.020
Asn	0.75	0.94	0.96	0.97	0.95	0.02	0.059
Orn	0.18	0.01	0.02	0.01	0.02	0.02	0.330
Hcy	0.38	0.38	0.38	0.35	0.39	0.00	0.726
GABA	0.04^b^	0.03^b^	0.04^b^	0.03^b^	0.05^a^	0.00	<0.001
FAA	69.75^b^	74.16^a^	73.55^a^	73.95^a^	74.01^a^	0.53	0.011
Total EAA	28.40^a^	24.51^b^	24.18^b^	24.27^b^	24.41^b^	0.52	0.013
Total NEAA	71.06^b^	75.48^a^	75.81^a^	75.72^a^	75.58^a^	0.57	0.007

a-d Means within a row with different superscript letters are significantly different (*P* < 0.05).

1 EAA: essential amino acids = lysine + methionine + threonine + leucine + isoleucine + arginine + valine + phenylalanine + histidine.

2 NEAA: Non-essential amino acids = Gly + Ala+ GABA+ Ser + Pro+ Asn + Orn + Asp+ Hcy + Gln + Glu + Arg + Tyr.

3 FAA: Flavor amino acids = Glu + Asp+ Ala+ Gly + Gln + Arg + Phe + Ile + Val + Tyr32 CK = basal diet (SBM); MML = SBM + 5 % marigold meal; MMH=SBM + 10 % marigold meal; MFL = SBM + 5 % fermented marigold meal; MFH=SBM + 10 % fermented marigold meal.

4 SEM = standard error of the mean (*n* = 6).

### 3.6 Muscle fatty acids profile

Table 2 presents the fatty acid composition of pectoralis major muscle in broilers following marigold meal supplementation. Compared with the CK group, the contents of saturated fatty acids (SFA) and PUFA were significantly increased, while the content of monounsaturated fatty acids (MUFA) was significantly decreased (*P* < 0.05). Specifically, the relative proportions of SFAs—including C12:0, C14:0, C16:0, C17:0, and C18:0—were significantly elevated in the treatment groups compared to CK (*P* < 0.05). Conversely, the proportions of MUFAs, particularly C16:1 and C18:1n7, were significantly reduced. The proportions of PUFAs, especially C18:2n6 and C18:3n3, were significantly increased in the marigold meal groups, whereas no significant difference was observed in C18:1n9c (*P* > 0.05). Regarding n-3 PUFAs, the MMH and MFL groups showed significantly higher contents compared with CK, while no significant differences were observed in the MML and MFH groups. Additionally, n-6 PUFA levels were markedly increased across all marigold meal-supplemented groups (*P* < 0.001). These findings suggest that dietary supplementation with fermented marigold meal is more effective in improving the fatty acid profile in the breast muscle of white feather broilers.

**Table 2:** Effect of marigold meal on fatty acids in breast muscle of white feather broilers at different doses (%).

Items	CK	MML	MMH	MFL	MFH	SEM	*P*-value
C12:0	0.04^c^	0.05^b^	0.06^ab^	0.07^a^	0.05^b^	0.002	<0.001
C14:0	0.37^c^	0.38^bc^	0.36^c^	0.42^ab^	0.44^a^	0.009	<0.001
C16:0	19.52^c^	19.56^c^	19.95^bc^	20.72^ab^	21.12^a^	0.194	0.003
C17:0	0.13^c^	0.14^bc^	0.13^c^	0.15^ab^	0.15^a^	0.003	0.002
C18:0	10.72^c^	11.84^abc^	12.33^a^	12.21^ab^	11.08^bc^	0.198	0.007
C20:0	0.07^b^	0.08^ab^	0.08^ab^	0.08^a^	0.08^ab^	0.001	0.072
SFA	30.86^b^	32.07^ab^	32.93^a^	33.66^a^	32.94^a^	0.304	0.009
C16:1	2.88^a^	2.38^b^	2.17^b^	2.27^b^	2.91^a^	0.09	<0.001
C18:1n7	2.80^a^	2.56^b^	2.33^c^	2.40^c^	2.23^c^	0.05	<0.001
C18:1n9C	23.52	22.61	21.05	23.48	23.56	0.34	0.063
C18:1n12	8.76	8.45	8.18	4.99	7.50	0.56	0.213
MUFA	37.97^a^	36.02^ab^	33.75^b^	33.15^b^	36.22^ab^	0.57	0.020
C18:2n6	16.63^c^	18.08^b^	19.19^ab^	19.72^a^	19.40^ab^	0.32	<0.001
C18:3n6	0.16^a^	0.15^ab^	0.14^b^	0.16^ab^	0.15^ab^	0.00	0.036
C18:3n3	0.52^c^	0.60^b^	0.70^a^	0.71^a^	0.75^a^	0.02	<0.001
C20:4n6	5.40^ab^	5.66^ab^	5.97^a^	5.76^a^	4.86^b^	0.12	0.023
C20:5n3	0.29^a^	0.24^b^	0.25^ab^	0.22^bc^	0.18^c^	0.01	<0.001
C22:5n3	0.78^ab^	0.88^ab^	0.94^a^	0.88^ab^	0.76^b^	0.02	0.027
C22:5n6	0.34^a^	0.34^a^	0.31^ab^	0.29^ab^	0.26^b^	0.01	0.006
C22:6n3	0.47	0.50	0.54	0.49	0.43	0.01	0.062
PUFA	24.63^b^	26.50^a^	28.07^a^	28.26^a^	26.82^a^	0.38	<0.001
P/S	0.79^c^	0.83^ab^	0.85^a^	0.84^ab^	0.81^bc^	0.00	0.002
n-3	2.07^c^	2.24^abc^	2.44^a^	2.31^ab^	2.13^bc^	0.03	0.003
n-6	22.55^b^	24.25^a^	25.63^a^	25.94^a^	24.68^a^	0.35	<0.001
n-6/n-3	10.85	10.78	10.51	11.21	11.54	0.13	0.107

a-c Means within a row with different superscript letters are significantly different (*P* < 0.05).

1 SFA: saturated fatty acid; MUFA: monounsaturated fatty acid; PUFA: polyunsaturated fatty acid; P/S: PUFA/SFA; n-3 PUFA: omega-3 polyunsaturated fatty acid; n-6 PUFA: omega-6 polyunsaturated fatty acid.

2 CK = basal diet (SBM); MML = SBM + 5 % marigold meal; MMH=SBM + 10 % marigold meal; MFL = SBM + 5 % fermented marigold meal; MFH=SBM + 10 % fermented marigold meal.

3 SFA = (C12:0 + C14:0 + C16:0 + C17:0 + C18:0 + C20:0).

MUFA = (C16:1 + C18:1n7 + C18:1n9C + C18:1n12).

PUFA = (C18:2n6 + C18:3n6 + C18:3n3 + C20:4n6 + C20:5n3 + C22:5n3 + C22:5n6 + C22:6n3).

n-3 = (C18:3n3 + C20:5n3 + C22:5n3 + C22:6n3).

n-6 = (C18:2n6 + C18:3n6 + C20:4n6 + C22:5n6).

4 SEM = standard error of the mean (n = 6).

### 3.7 VOCs analysis

**Fig. 2:**
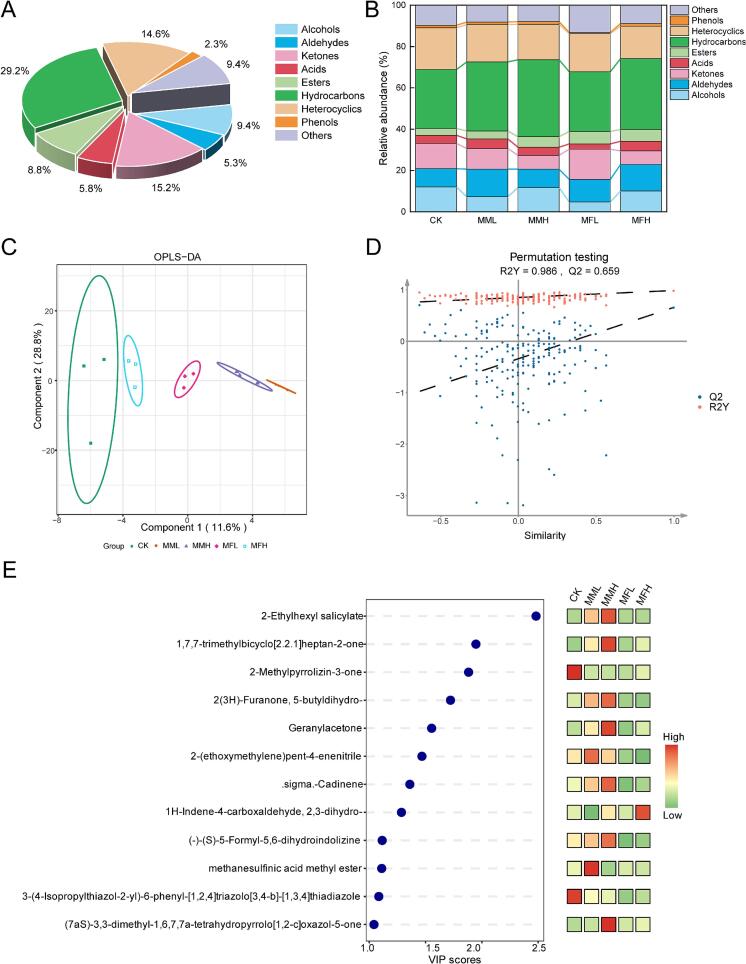
Effects of marigold meal on volatile compounds in deep-fried broiler breast muscle. (A) Types of volatile compounds; (B) relative contents of volatile compounds; (C) OPLS-DA; (D) results of the OPLS-DA permutation test; and (E) VIP scores. Component 1 denotes the first principal component, Component 2 denotes the second principal component, and the ellipses represent the 95 % confidence intervals.

To investigate the impact of fermented marigold diets on meat flavor, we analyzed the volatile compounds in fried chicken breast samples from different dietary groups. GC–MS identified 171 VOCs across all samples (Supplementary Fig. S2). These compounds were primarily classified as alcohols (9.4 %), aldehydes (5.3 %), ketones (15.2 %), acids (5.8 %), esters (8.8 %), hydrocarbons (29.2 %), heterocyclic compounds (14.6 %), phenols (2.3 %), and others (9.4 %), with hydrocarbons and ketones being the most abundant classes (Fig. 2A). Notably, dietary supplementation with marigold meal significantly altered the volatile flavor profile of chicken meat. Broilers receiving marigold meal exhibited higher overall levels of flavor-related compounds, including aldehydes, hydrocarbons, and ketones, in their fried breast meat compared with the CK group. The MML and MFH groups contained the highest total aldehyde levels, the MMH and MFH groups had the highest hydrocarbon levels, and the MFL group showed the greatest abundance of ketones (Fig. 2B).

Orthogonal partial least squares discriminant analysis (OPLS-DA) was further conducted to differentiate the groups according to their metabolic profiles. As shown in Fig. 2C, the volatile compound profiles of the CK group were clearly separated from those of the marigold meal-treated groups. To avoid model overfitting, a permutation test was applied to verify the validity of the model during its construction. The results of the permutation test for OPLS-DA are shown in Fig. 2D, with R^2^Y and Q^2^ values of 0.986 and 0.659, respectively. As the retention rate in the permutation test decreased, the R^2^ and Q^2^ values of the stochastic models also declined, indicating that the original model was not overfitted. This finding confirms that the differences in metabolite groupings identified by OPLS-DA were statistically significant. Based on variable importance in projection (VIP) scores and significance screening, 12 compounds were identified as potential intergroup aromatic markers (VIP > 1.0, FC > 1.2, *P* < 0.05) (Fig. 2E). The identified compounds included 2-ethylhexyl salicylate, 1,7,7-trimethylbicyclo[2.2.1]heptan-2-one, 2-methylpyrrolizin-3-one, 2(3H)-furanone, 5-butyldihydro-, Geranylacetone, 2-(ethoxymethylene)pent-4-enenitrile, σ-cadinene, 1H-indene-4-carboxaldehyde, 2,3-dihydro-, (−)-(*S*)-5-formyl-5,6-dihydroindolizine, methanesulfinic acid methyl ester, 3-(4-isopropylthiazol-2-yl)-6-phenyl-[1,2,4]triazolo[3,4-b]-[1,3,4]thiadiazole, and (7aS)-3,3-dimethyl-1,6,7,7a-tetrahydropyrrolo[1,2-*c*]oxazol-5-one. Ketones were also identified as the major potential markers, accounting for 33.3 % of all markers, highlighting the importance of the Maillard reaction and lipid oxidation products in meat aroma composition. In addition, several flavor-related compounds were significantly more abundant in the MMH group than in the CK group. For example, 2(3H)-furanone, 5-butyldihydro- (a lactone), and Geranylacetone (a terpenoid ketone) were significantly elevated in the marigold-supplemented diet groups, with the unfermented marigold group showing particularly high levels. These two compounds are known to impart sweet, milky flavors and floral (magnolia-like) aromas to foods, respectively. Therefore, marigold meal supplementation enhanced the sensory qualities of fried chicken by increasing the abundance of such pleasant aroma compounds.

### 3.8 Intestinal microbiome analysis

**Fig. 3:**
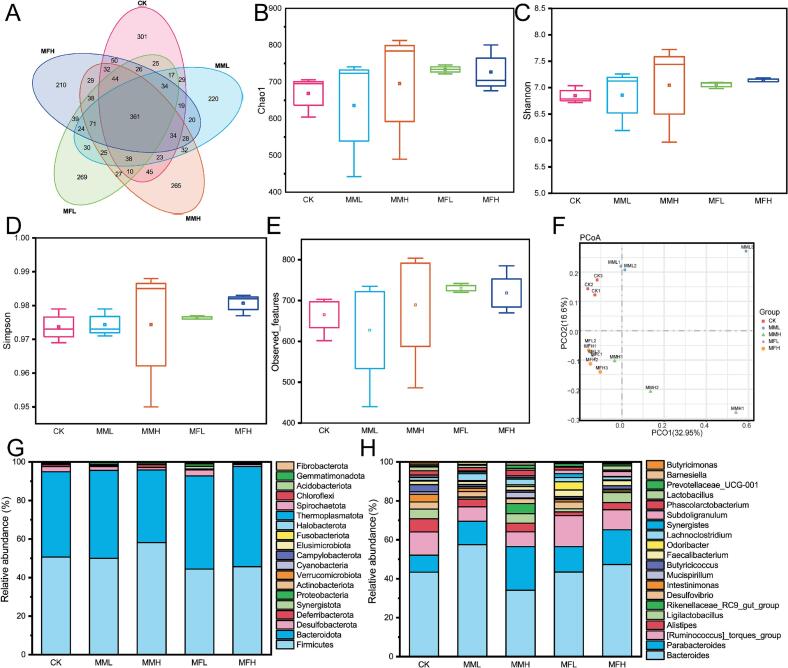
Effect of marigold meal on diversity and species composition of intestinal microbial community in broilers. (A) Venn diagram analysis of gut microbiota sequencing; (B-E) Alpha diversity index of gut microbiota; (F) PCoA plot; (G, H) relative abundance of top 20 phyla and genus level species within gut microbiota. Groups: CK (control), MML (5 % marigold meal), MMH (10 % marigold meal), MFL (5 % fermented marigold meal), MFH (10 % fermented marigold meal).

Fig. 3A presents a Venn diagram illustrating the distribution of amplicon sequence variants (ASVs) among treatment groups. A total of 2599 ASVs were identified, with 361 ASVs shared across all five groups. Unique ASVs were detected in each group, with 1088 in CK, 1005 in MML, 1102 in MMH, 1078 in MFL, and 1059 in MFH, indicating that marigold meal supplementation had a considerable impact on the cecal microbial community composition. Alpha diversity metrics—including the Chao1, Shannon, Simpson, and Observed species indices (Fig. 3B–E)—were used to evaluate within-sample microbial richness and diversity. Although no significant differences were observed compared with the CK group (*P* > 0.05), a trend toward increased diversity was noted in all marigold meal-supplemented groups. Beta diversity analysis based on Bray–Curtis distances was performed to evaluate inter-group variation in microbial community structure. As shown in Fig. 3F, the MML, MMH, MFL, and MFH groups exhibited significant separation from the CK group, suggesting that marigold meal supplementation, particularly in fermented forms, altered the overall microbial community composition. Taxonomic analysis of microbial composition at the phylum and genus levels is shown in Fig. 3G and H, respectively. At the phylum level, Firmicutes, Bacteroidota, Desulfobacterota, Deferribacterota, and Synergistota were dominant. Notably, the relative abundance of *Bacteroidota* increased from 44 % in the CK group to 52 % in the MFH group. At the genus level, predominant taxa included *Bacteroides*, *Parabacteroides*, *[Ruminococcus]_torques_group*, *Alistipes*, and *Ligilactobacillus*. For instance, *Bacteroides* abundance rose from 43 % in CK to 47 % in MFH, indicating enhanced proliferation of beneficial bacteria in response to fermented marigold meal supplementation.

### 3.9 Discriminative cecal microbiota and functional profiles revealed by LEfSe and PICRUSt2 analysis

**Fig. 4:**
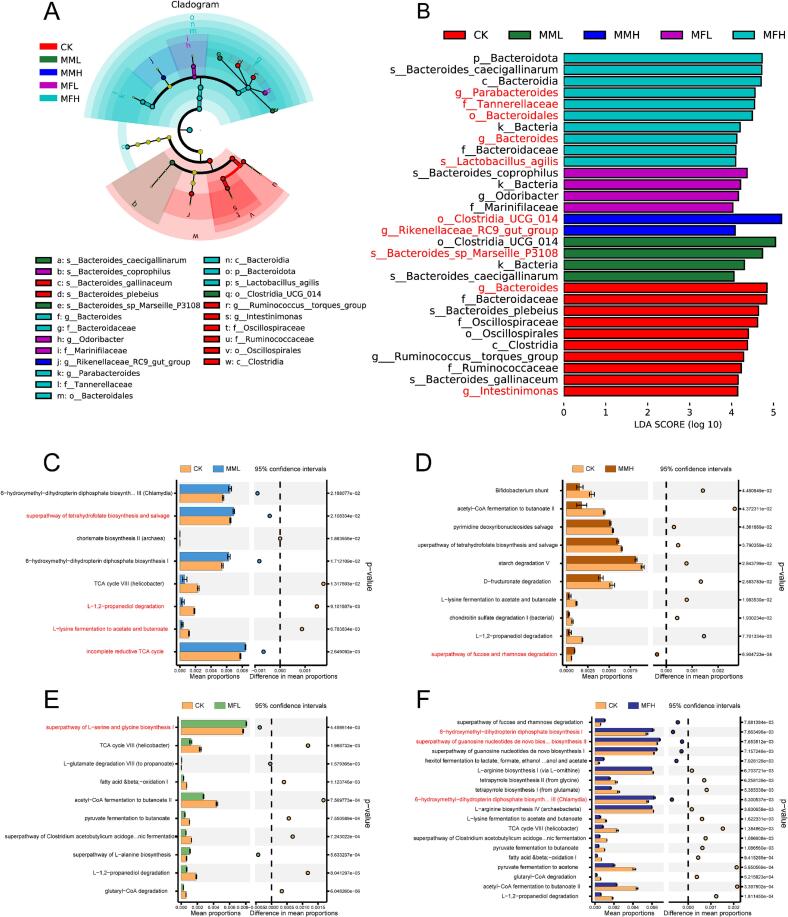
Combined effect size determination (LEfSe) and linear discrimination (LDA) analysis of the microbiota of the caecum of broilers. (A) From metagenomic data, circle size reflects taxon abundance. (B) Branch diagram of LEfSe analysis. (C—F) Pathway analysis of difference at L3 level between CK group and MML, MMH, MFL and MFH groups.

To further explore the effects of different marigold meal treatments on specific bacterial taxa, linear discriminant analysis effect size (LEfSe) was conducted using an LDA threshold of 4.0 to identify significantly enriched biomarkers among groups. As shown in Fig. 4A, a total of six taxonomic clades across five phyla were identified, with the majority of differential taxa derived from the MFH group, suggesting that fermented marigold meal exerted a pronounced modulatory effect on the cecal microbiota. LEfSe results revealed that *Bacteroides* and *Intestinimonas* were significantly enriched in the CK group (LDA > 4), while *Bacteroides_sp_Marseille_P3108* was enriched in the MML group. In the MMH group, the dominant taxa included *Clostridia_UCG_014* and *Rikenellaceae_RC9_gut_group* (LDA > 4). The MFL group exhibited enrichment of *Bacteroides_coprophilus*. In the MFH group, key discriminatory taxa included *Parabacteroides*, *Tannerella*, and *Lactobacillus_agilis*, all with LDA scores exceeding 4 (Fig. 4B). These findings indicate that fermented marigold meal, particularly in the MFH group, promoted the colonization of beneficial bacteria, thereby enhancing the abundance of functionally relevant microbial taxa in the broiler cecum.

Functional prediction of the cecal microbiota was performed using PICRUSt2 based on 16S rRNA sequencing data, and metabolic pathway enrichment was annotated according to the Kyoto Encyclopedia of Genes and Genomes (KEGG) database at level 3 (L3). As shown in Fig. 4C, the MML group exhibited upregulation of the *6-hydroxymethyl-dihydropterin phosphate biosynthesis III (Chlamydia)* pathway, the *superpathway of tetrahydrofolate biosynthesis and salvage*, and the *incomplete reducing TCA cycle*, while pathways such as *L-1,2-propanediol degradation* and *l-lysine fermentation to acetate and butanoate* were downregulated compared with the CK group. In the MMH and MFL groups, Fig. 4D and E shows upregulation of the *superpathway of fucose and rhamnose degradation* and the *superpathway of l-serine and glycine biosynthesis I*, respectively. The MFH group demonstrated significant enrichment of several biosynthetic pathways, including *6-hydroxymethyl-dihydropterin diphosphate biosynthesis I*, the *superpathway of guanosine nucleotides* de novo *biosynthesis II*, and *6-hydroxymethyl-dihydropterin phosphate biosynthesis III (Chlamydia)* (Fig. 4F). These results highlight distinct differences in functional metabolic pathway profiles among the marigold meal intervention groups, suggesting that marigold meal—particularly in fermented form—may modulate energy metabolism and weight regulation in broilers by altering the abundance of specific microbial metabolic pathways.

### 3.10 Short-chain fatty acid analysis (SCFA)

**Fig. 5:**
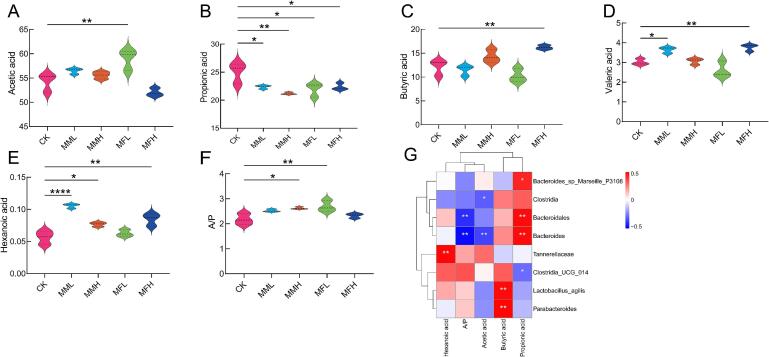
Effect of marigold meal on SCFAs content in caecum of broilers. (A) Acetic acid; (B) Propionic acid; (C) Butyric acid; (D) Valerate acid; (E) Hexanoic acid; (F) Acetic/ Propionic acid; (G) Heat map of correlation analysis between SCFAs and intestinal microbes Spearman.

To evaluate the impact of marigold meal supplementation on microbial metabolism in the broiler cecum, concentrations of key SCFAs—including acetic acid, propionic acid, butyric acid, valeric acid, and hexanoic acid—along with the acetic acid to propionic acid ratio (A/P), were determined. As shown in Fig. 5A–F, total SCFA concentrations were significantly increased in all marigold meal-treated groups compared to the CK group (*P* < 0.05), with the MFH group exhibiting the most pronounced effect (*P* < 0.05). Interestingly, despite the general increase in SCFAs, the concentration of propionic acid decreased significantly following marigold meal supplementation (*P* < 0.05), indicating a shift in microbial fermentation patterns. Spearman correlation heatmap analysis (Fig. 5G) revealed significant associations between SCFA levels and specific microbial taxa. Acetic acid was negatively correlated with *Bacteroides* and *Clostridia*, whereas propionic acid showed a positive correlation with *Bacteroides*, *Bacteroidales*, and *Bacteroides_sp_Marseille_P3108*, and a negative correlation with *Clostridia_UCG_014*. Additionally, butyric acid levels were positively correlated with *Parabacteroides* and *Lactobacillus agilis*. These results suggest that marigold meal, particularly in fermented form, not only alters SCFA production in the cecum but also modulates the abundance of key bacterial taxa involved in SCFA metabolism.

### 3.11 Correlation analysis between gut microbiota and muscle metabolites

**Fig. 6:**
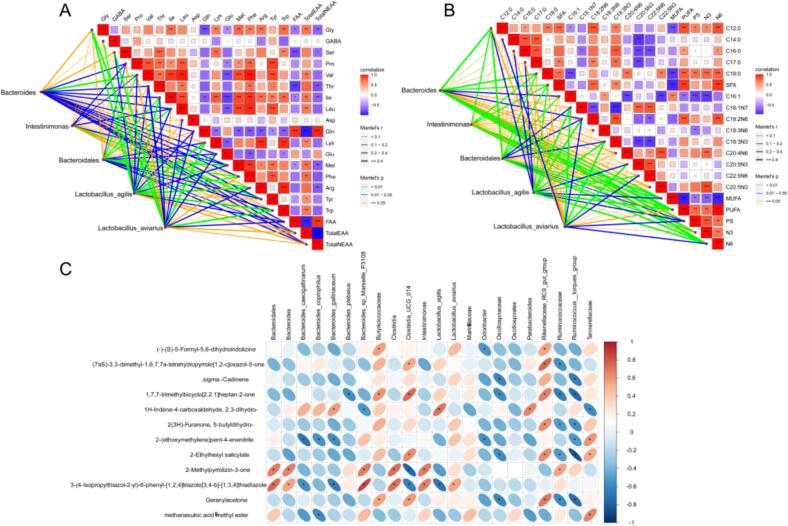
Correlation analysis of amino acids, fatty acids and intestinal microorganisms. (A) Mantel test analysis chart of chicken differential flora and amino acids;(B) Mantel test analysis chart of chicken differential flora and fatty acids; (C) Heatmap showing the correlations between differential intestinal taxa and key flavor compounds in chicken meat.

To explore the relationship between cecal microbial composition and host metabolic profiles, Mantel tests were performed to assess correlations between differential microbial taxa and amino acid or fatty acid contents in broiler breast muscle. As shown in Fig. 6A, *Bacteroidales* exhibited strong positive correlations with essential amino acids, including methionine (Met), leucine (Leu), phenylalanine (Phe), and isoleucine (Ile). *Lactobacillus* was positively associated with Ile, lysine (Lys), glycine (Gly), glutamic acid (Glu), and GABA. In addition, both total EAA and total NEAA were mainly correlated with *Bacteroidales* and *Bacteroides*. Regarding fatty acids (Fig. 6B), *Bacteroides* were positively associated with stearic acid (C18:0), PUFA, n-6 PUFA, SFA, n-3 PUFA, and the PUFA/SFA ratio (P/S). *Intestinimonas* showed strong positive correlations with MUFA, n-6 PUFA, and PUFA. *Lactobacillus* was linked to several unsaturated fatty acids, including C18:1n7, C18:2n6, C18:3n3, and C20:5n3. Pearson's correlation analysis was performed to examine the associations between differential intestinal flora and the concentrations of key flavor compounds in different treatment groups. As shown in Fig. 6C, in the CK group, the dominant taxa *Ruminococcaceae* and *Ruminococcus_torques_group* generally exhibited significant negative correlations (*P* < 0.05) with these aroma compounds. In contrast, in the MML and MMH groups, the dominant taxa *Clostridia_UCG_014* and *Rikenellaceae_RC9_gut_group* exhibited significant positive correlations (*P* < 0.05) with most volatile compounds. These findings suggest that unfermented marigold meal may promote the accumulation of flavor precursors or volatile products in the host by enriching specific intestinal taxa. Notably, the dominant taxa in the fermented marigold meal groups exerted weaker effects on volatile compounds and may influence flavor through more direct pathways that are less dependent on the intestinal microbiota.

## 4 Discussion

In this study, through the determination of the components of marigold meal and its fermentation products, it was found that fermentation significantly increased the total flavonoid, total sugars, total phenol and total protein contents in the residue, which may be due to the fact that lactic acid bacteria destroyed plant cell walls and released bound phenols and polysaccharides by secreting hydrolytic enzymes such as cellulase and protease during fermentation. This process is supported by SEM and XRD analyses, which show destruction of fiber structure and change of crystallinity. This finding echoes the fermentation enrichment effect proposed by Manivannan et al. (Manivannan, Narasegowda, & Prakash, 2021), suggesting that fermented marigold meal has potential as a functional feed additive.

In terms of growth performance, the addition of marigold meal in the diet had no significant effect on the growth performance of white feather broilers (*P* > 0.05). This discovery is consistent with S. Wang's findings were consistent, reporting that 0.075 %, 0.15 %, 0.30 % and 0.60 % marigold extract also had no significant regulatory effect on growth curve and feed conversion efficiency of broilers(S. Wang et al., 2017). In addition, the application of marigold meal resulted in a weight gain of approximately 500 g compared to studies on walnut meal replacement protein, showing its potential to improve production performance(Jiang et al., 2024). Meat quality is typically evaluated by measuring pH, color, water-holding capacity (WHC), and shear force(Chang et al., 2023). WHC reflects the ability of muscle tissue to retain moisture and is a key determinant of sensory attributes such as juiciness, tenderness, and nutritional value(S. Wang et al., 2017). This study shows that, marigold meal supplementation had no significant effects on drip loss, pH, meat color (L*, a*, b*), shear force, crude fat, crude protein, or moisture content in the breast muscle of broilers (*P* > 0.05), indicating that marigold meal is a safe and stable feed additive with minimal impact on fundamental meat quality traits. These findings are consistent with previous research on dietary supplementation with lactic acid bacteria or prebiotics, which improved certain meat quality attributes without altering meat color (Liu et al., 2023).

To further evaluate the nutritional value of broiler breast muscle, amino acid and fatty acid profiles were determined using gas chromatography–mass spectrometry (GC–MS). Muscle amino acid composition is closely associated with both the flavor and nutritional quality of meat(Moeller et al., 2010). EAAs are key determinants of protein biological value, whereas FAAs contribute directly to taste and palatability(F. Yang et al., 2020). In the present study, marigold meal supplementation led to an increase in NEAAs, particularly glutamine and glutamic acid, as well as in total FAAs (including Glu, Asp, Ala, Gly, Gln, Arg, Phe, Ile, Val, and Tyr) in chicken breast muscle. Conversely, a reduction in total EAA content was observed. This shift may be attributed to bioactive components such as polysaccharides present in marigold meal, which could modulate intestinal microbial metabolism or influence nitrogen utilization efficiency in the host. Previous studies have shown that dietary supplementation with polysaccharide–zinc complexes can regulate amino acid metabolism by enhancing antioxidant capacity, potentially promoting the biosynthesis of NEAAs(Wassie, Duan, Xie, Wang, & Wu, 2022). Flavor-related amino acids such as Glu, Ser, Ala, Gly, and Arg are known to serve as precursors for volatile compounds that enhance meat taste(Qi, Men, Wu, & Xu, 2019). For example, dietary supplementation with 150 mg/kg glycerol monolaurate significantly increased the levels of Lys, Asp, Glu, Tyr, umami amino acids, and total amino acids in broiler breast muscle(Feng et al., 2021). Furthermore, fermentation-derived microorganisms may partially degrade or utilize EAAs during microbial metabolism, leading to lower final EAA content in both feed and animal tissues(Xiao, Sun, Xin, Xu, & Du, 2023). The patterns observed in this study suggest that marigold meal—particularly in fermented form—may exert similar effects through both direct nutrient transformation and modulation of host–microbiota interactions.

Another key determinant of meat flavor and nutritional quality is the composition and content of intramuscular fatty acids(Wood et al., 2008). In poultry, unsaturated fatty acids typically predominate over SFAs, with PUFAs accounting for nearly half of total fatty acids(Xu et al., 2024). In the present study, C16:0, C18:0, C18:1, and C18:2 together accounted for approximately 70 % of the total fatty acids detected in broiler breast muscle. Fatty acids also contribute to meat flavor through oxidation products. For example, C18:1 can oxidize to form heptanal, octanal, and nonanal, while C18:2 can generate hexanal, (E)-2-octenal, 1-octen-3-ol, and 2-pentylfuran, compounds associated with desirable aroma profiles(Van Ba, Amna, & Hwang, 2013). In this study, supplementation with marigold meal significantly increased the contents of both SFAs and PUFAs—including C18:2n-6, C18:3n-3, and C20:4n-6—in breast muscle. As a result, the PUFA/SFA ratio, as well as levels of n-3 and n-6 fatty acids, were elevated. Meanwhile, the proportion of C18:1n-7 was reduced, while C18:1n-9 and the n-6: n-3 ratio remained unchanged. These results are consistent with previous reports on the fatty acid profile of egg yolk following marigold supplementation(Ayse & Rahim, 2014). The underlying mechanism may be twofold: first, marigold meal is naturally rich in SFAs and PUFAs, which are readily deposited in muscle tissue after ingestion(Kanakri, Carragher, Hughes, Muhlhausler, & Gibson, 2018); second, marigold components may modulate lipid metabolism, particularly by downregulating stearoyl-CoA desaturase (SCD), the enzyme responsible for converting C16:0 and C18:0 to C18:1n-7. Additionally, the stability of C18:1n-9 and the n-6: n-3 ratio may reflect intrinsic biological regulation aimed at maintaining cell membrane integrity and lipid homeostasis(Ding, Yuan, Huang, Jiang, & Qian, 2021). Taken together, these findings suggest that marigold meal supplementation can improve the fatty acid composition of broiler breast meat, thereby enhancing its nutritional value through both dietary lipid enrichment and modulation of endogenous lipid metabolism.

This study demonstrated that dietary supplementation with marigold meal significantly enhanced the flavor chemistry of broiler breast muscle. Broiler breast muscle from the marigold meal group contained higher levels of several aroma-active volatiles compared with the basal diet group, consistent with an improved intramuscular lipid profile (e.g., elevated PUFA content). Ketones (e.g., 2-pentanone, terpene ketones) in chicken meat are mainly generated during high-temperature cooking through the Maillard reaction, lipid oxidation, and amino acid catabolism (Zhang, Hu, Wang, Kong, & Chen, 2021). In the present study, the essential amino acid content of breast muscle from broilers in the marigold meal group decreased, which may have promoted the formation of Maillard-derived ketones via enhanced Strecker degradation of amino acids. Meanwhile, aldehydes (e.g., hexanal, heptanal, nonanal) were primarily generated via oxidation of unsaturated fatty acids and Strecker degradation of amino acids(C. Chang, Wu, Zhang, Jin, & Wang, 2020). These aldehydes are characterized by low odor thresholds and high aroma contributions, making them important flavor-active compounds in meat. The marigold meal group, particularly the fermented marigold meal group, significantly increased the PUFA content (especially n-3 fatty acids) in the pectoral muscle, which may have resulted in greater production of lipid oxidation-derived aldehydes during frying(Zhao et al., 2024). This observation is consistent with recent reports that plant-based feed additives can enrich dietary unsaturated fatty acids and enhance meat flavor. For instance, feeding broilers dendrobium leaves has been shown to elevate n-3 PUFA levels in breast muscle, accompanied by significant increases in key volatiles such as hexanal, glutaraldehyde, and heptanal, thereby improving overall meat aroma(Zhao et al., 2024). Similarly, dietary curcumin supplementation increased the production of aldehyde aroma compounds (e.g., hexanal, octanal, nonanal) in chicken meat, thereby intensifying meat flavor(Shu et al., 2024). In the present study, marigold meal supplementation not only increased the aforementioned lipid oxidation-derived aldehydes but also led to the emergence of distinctive terpene and lactone aromas (e.g., Geranylacetone, δ-cadinene, furanones) in chicken meat. This could be attributed to the deposition of marigold-derived bioactive constituents (e.g., carotenoids, terpenoids) and their thermal degradation products in muscle tissue. Notably, Geranylacetone—a carotenoid cleavage product—was elevated in the marigold meal group, likely due to terpene aroma formation via the thermal cleavage of lutein and zeaxanthin, carotenoid-rich constituents of marigold. For example, 2(3H)-furanone, 5-butyldihydro- (with a milky-sweet aroma) was detected exclusively in the marigold-treated group, suggesting that marigold meal supplementation introduced novel flavor precursors absent in the control group(Goel et al., 2024). Chicken meat from marigold meal-fed broilers exhibited more intense sweet, creamy, and floral notes due to elevated furanones and terpenes, which complemented fatty aldehydes to enhance overall meat flavor. However, although elevated unsaturated fatty acids can increase the formation of oxidized volatiles, antioxidant constituents in marigolds may modulate the lipid oxidation process. Dietary polyphenols are known to inhibit excessive lipid oxidation and reduce the formation of odorants. Previous studies have reported that tea polyphenols significantly reduce thiobarbituric acid (TBA) values and certain aldehydes in cooked chicken meat, thereby diminishing fishy odors(Ning et al., 2025). In the present study, the fermented marigold meal group exhibited higher total phenolic content and antioxidant activity, which may have balanced the levels of volatiles generated from lipid oxidation during cooking. In conclusion, fermented marigold meal, as a feed additive, can improve chicken flavor by enhancing the deposition of unsaturated fatty acids in muscle and directly supplying both flavor precursors and antioxidants.

The cecum is a crucial intestinal site in broilers, hosting a highly diverse and metabolically active microbial community that plays essential roles in nutrient digestion and absorption, energy homeostasis, and immune system development. Microbial richness in the cecum is closely associated with the stability of the intestinal microenvironment(Le Chatelier et al., 2013). Therefore, we investigated the effects of marigold meal supplementation on cecal microbiota composition. Our results revealed that the cecum harbored the highest microbial diversity within the gastrointestinal tract, with dominant phyla including *Firmicutes*, *Bacteroidota*, and *Proteobacteria*, among which *Firmicutes* predominated (Zhang et al., 2023). Notably, fermentation treatment significantly increased the relative abundance of *Bacteroides*, a genus known for its critical role in polysaccharide degradation, immune modulation, and microbiota balance(Shan et al., 2025). LEfSe analysis further identified *Parabacteroides* and *Lactobacillus* as discriminatory taxa in the MFH group, both of which were strongly positively correlated with butyric acid levels. As a key SCFA, butyric acid serves as the primary energy source for colonic epithelial cells, enhances intestinal barrier integrity, and mitigates endotoxin translocation, thereby contributing to systemic anti-inflammatory effects and improved muscle metabolism(Hertli & Zimmermann, 2022). Previous studies have shown that broiler populations with higher cecal butyrate concentrations exhibit enhanced muscle development and stronger disease resistance(Li, Jin, & Fan, 2022). Moreover, KEGG-based functional prediction revealed that marigold meal supplementation upregulated metabolic pathways involved in folate and guanosine nucleotide biosynthesis, suggesting improved microbial contributions to host energy metabolism (Liu et al., 2023). Combined with Mantel correlation analysis, we observed significant positive associations between beneficial taxa (e.g., *Lactobacillus*, *Bacteroidales*) and functional nutrient components such as GABA, Glu, and PUFAs. Taken together, these findings suggest that marigold meal, particularly in fermented form, improves muscle nutritional quality through a synergistic mechanism involving gut microbiota modulation, microbial metabolic activation, and enhanced nutrient deposition—forming a functional axis of “microbiota–metabolic pathway–muscle nutrition”.

Despite extensive analyses, several limitations remain. Storage stability under chilled conditions and structured sensory testing were not performed, so links between antioxidant and shelf-life or perception are inferential. Chemical data were largely relative; absolute targeted quantitation and aroma-activity confirmation (GC-O/OAV) were not conducted. Evidence for microbiota mediation is associative, lacking SCFA profiling and interventional or histological validation. Future work should integrate storage and sensory trials with targeted quantitation and GC-O/OAV, incorporate SCFA-based and interventional studies to establish causality, and assess generalizability and scalable, standardized fermentation including safety and techno-economics.

## 5 Conclusions

In conclusion, dietary supplementation with fermented marigold meal significantly improved the nutritional composition of broiler muscle and favorably modulated the gut microbiota. Broilers receiving marigold supplementation exhibited higher levels of key amino acids (e.g., glutamic acid, γ-aminobutyric acid) and n-3 polyunsaturated fatty acids, increased abundances of beneficial cecal bacteria such as *Lactobacillus* and *Parabacteroides*, and elevated butyric acid levels. Flavor analysis revealed that fried breast meat from marigold-fed chickens contained higher concentrations of volatile flavor compounds. These findings suggest that fermented marigold meal may improve meat quality through microbiota-mediated mechanisms. Mechanistically, fermentation increased the bioactive nutrient content of marigold meal, and dietary inclusion of fermented marigold meal could remodel the intestinal microbiota, thereby enhancing metabolite production (e.g., SCFAs, GABA) and promoting the deposition of functional nutrients and flavor precursors in muscle tissue. The results indicate that fermented marigold residues can serve as a sustainable functional feed additive in poultry production to enhance both meat nutrition and flavor. This approach offers a novel strategy for the high-value utilization of agricultural by-products and for reducing reliance on synthetic additives, which is critical for sustainable poultry production and meat quality improvement.

## CRediT authorship contribution statement

**Zezhu Du:** Writing – original draft, Formal analysis, Data curation. **Yan Shen:** Validation, Methodology, Data curation. **Jinya Dong:** Validation, Methodology, Data curation. **Siyu Zhou:** Writing – review & editing. **Yuanfeng Chen:** Writing – review & editing. **Huiqing Luo:** Writing – review & editing. **Shikuan Zhao:** Writing – review & editing. **Zhiyu Li:** Supervision. **Cheng Gong:** Supervision. **Lihui Yu:** Supervision. **Xiaocui Du:** Validation, Software. **Tianjun Li:** Validation, Software. **Yunfei Ge:** Funding acquisition, Conceptualization. **Ruijuan Yang:** Funding acquisition, Conceptualization. **Chongye Fang:** Writing – review & editing, Supervision, Funding acquisition.

## Ethics statement

The animal experimentation protocol adhered to the guidelines and approvals set forth by the Life science Ethics Committee, Yunnan Agricultural University (approved # APYNAU 202505047).

## Declaration of competing interest

The authors declare that they have no known competing financial interests or personal relationships that could have appeared to influence the work reported in this paper.

## Data Availability

Data will be made available on request.

## Appendix A Supplementary data

Supplementary material Image 1
