# Supplementary material for: Fermented marigold meal enhances muscle nutrition and flavor in white feathered broilers via gut microbiota modulation
Source: Food Chem X. 2025 Oct 14;31:103165. doi: 10.1016/j.fochx.2025.103165 (PMC12554067; doi:10.1016/j.fochx.2025.103165)
Supplement: Supplementary file 1 — Supplementary material [file mmc1.docx]

**Supplementary Materials**

**Fermented marigold meal enhances muscle nutrient composition by modulating gut microbiota in white feather broilers**

**Authors**

Zezhu Du^a, b,1^, Yan Shen^a, b,1^, Jinya Dong^a, b^, Siyu Zhou^a, b^, Yuanfeng Chen^a, b^, Huiqing Luo^a, b^, Shikuan Zhao^a, b^, Zhiyu Li^a, b^, Cheng Gong^b, c^, Lihui Yu^b^, Xiaocui Du^b, c^, Tianjun Li^c^, Yunfei Ge^a, b, *^, Ruijuan Yang^a, b, *^, Chongye Fang^a, b, *^

**Affiliations**

^a^ College of Food Science and Technology, Yunnan Agricultural University, Kunming 650201, China

^b^ Yunnan Research Center for Advanced Tea Processing, Yunnan Agricultural University, Kunming 650201, China

^c^ College of Agronomy and Biotechnology, Yunnan Agricultural University, Kunming 650201, China

*** Corresponding authors at:** College of Food Science and Technology, Yunnan Agricultural University, No. 452, Fengyuan Road, Panlong District, Kunming 650201, China (C. Fang).

E-mail addresses*:* [ffccyy2@163.com](mailto:ffccyy2@163.com) (C. Fang); [Helen_4680@163.com](mailto:Helen_4680@163.com) (R. Yang); [18345965861@163.com](mailto:18345965861@163.com) (Y. Ge).

^1^ These authors contributed equally to this work.

**Supplementary Figures**

**
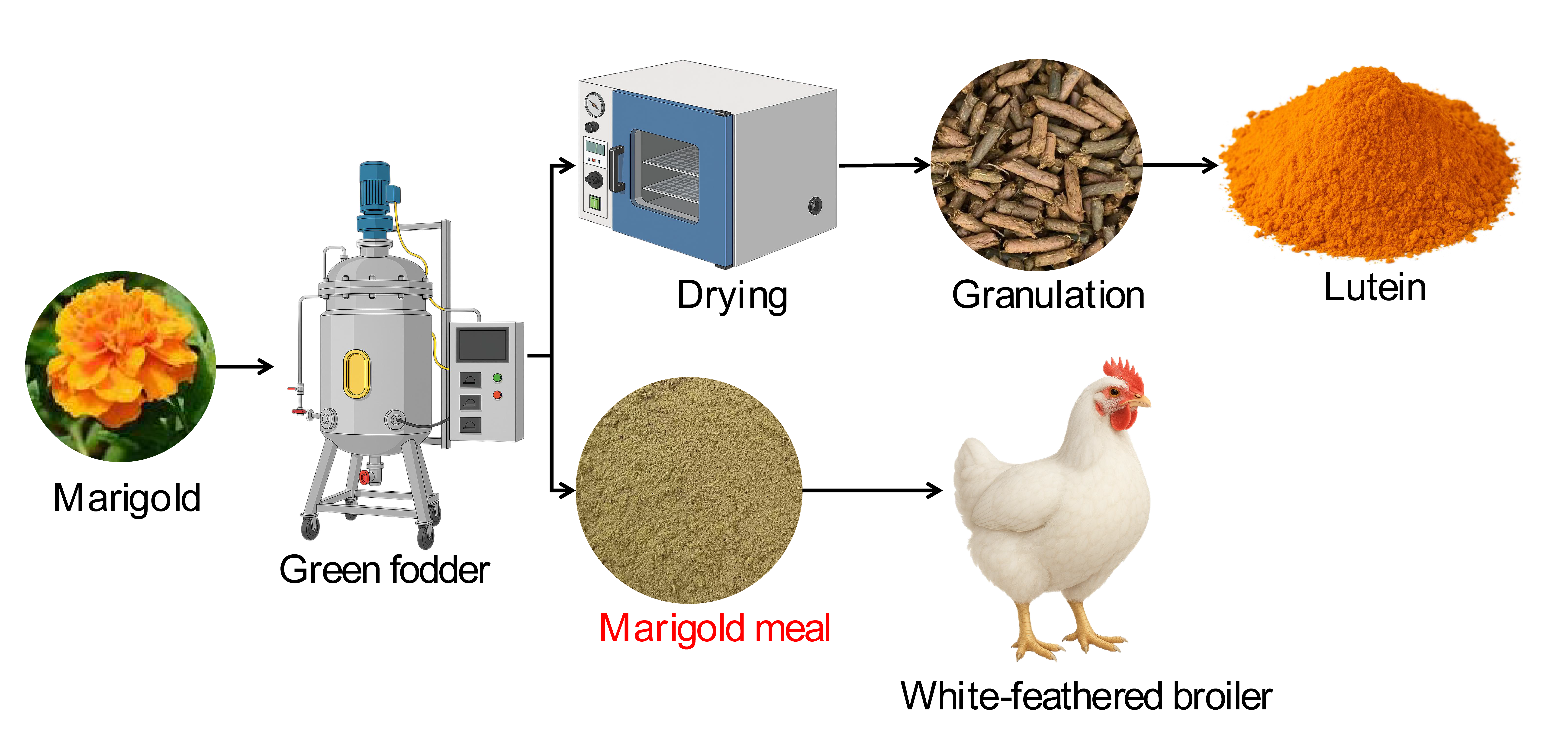
**

**Supplementary Fig. S1.** Preparation process of marigold meal. First, fresh marigold flowers are harvested and crushed. Subsequently, the crushed material is stacked in silage tanks to initiate fermentation. After fermentation, a portion of the water is released as fermentation wastewater, and additional moisture is removed from the marigold material through pressing. Finally, the marigold meal is dried and granulated to facilitate storage and subsequent lutein extraction.

**
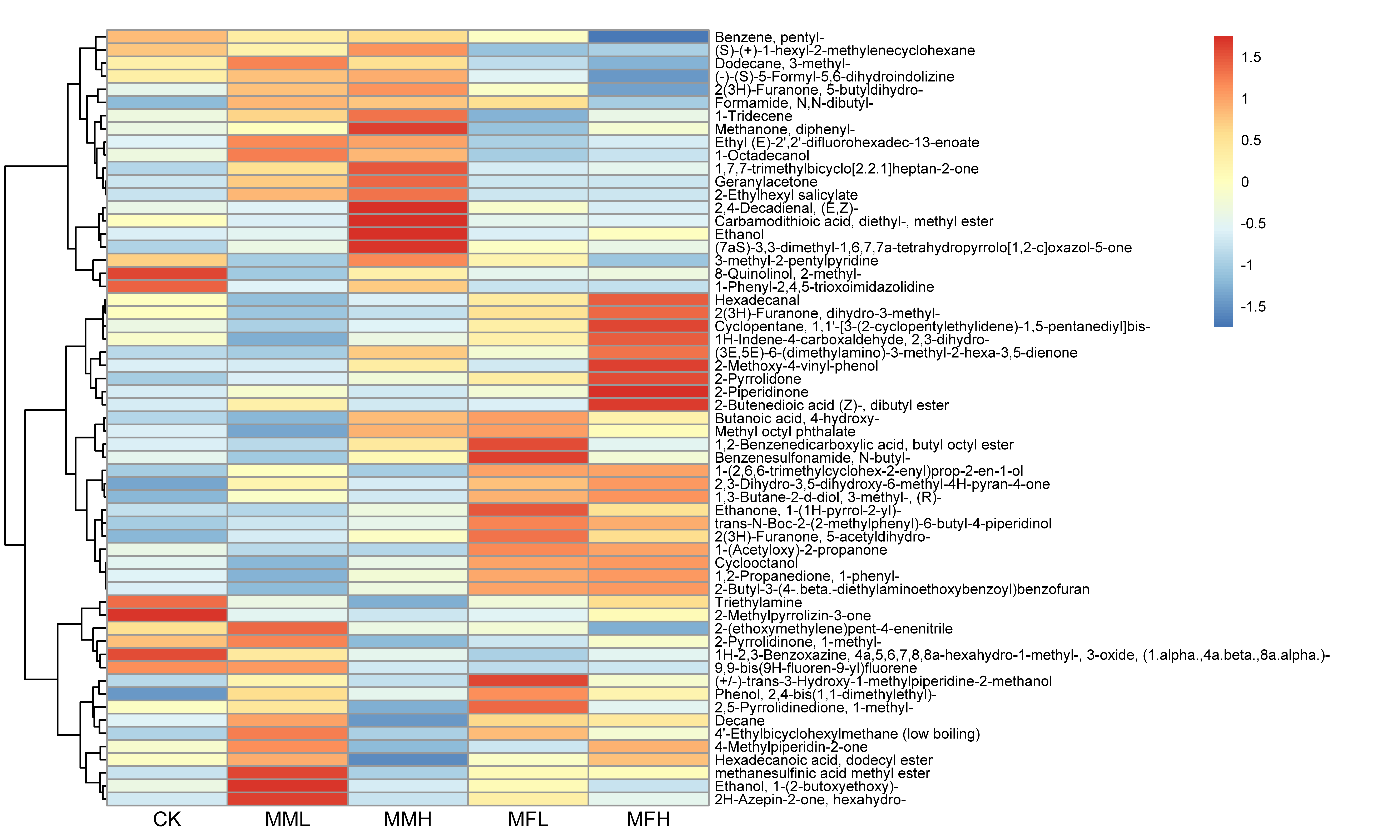
**

**Supplementary Fig. S2.** Heat map of total volatile compounds content.

**Supplementary Tables**

**Table S1** Composition and nutrient level of the basic diet

| **Raw material composition /%** | 1–21 days of age | 22–42 days of age |
| --- | --- | --- |
| Corn | 58.50 | 60.10 |
| Soybean oil | 3.04 | 4.50 |
| Soybean meal | 28.00 | 30.00 |
| Fermented soybean meal | 5.00 | – |
| Sodium chloride | 1.60 | 1.35 |
| Threonine | 0.10 | 0.09 |
| Salt | 0.22 | 0.26 |
| Lysine | – | 0.10 |
| Methionine | 0.14 | 0.15 |
| Calcium biphosphate | 1.40 | 1.45 |
| Premix ^1^ | 2.00 | 2.00 |
| Total | 100.00 | 100.00 |
| **Nutrient level /%** |  |  |
| Metabolizable energy ^2^ /MJ kg^-1^ | 12.33 | 12.76 |
| Crude protein | 21.04 | 18.97 |
| Crude fat | 5.60 | 7.06 |
| Calcium | 1.00 | 0.90 |
| Methionine | 0.43 | 0.40 |
| Lysine | 1.08 | 1.01 |
| Total phosphorus | 0.65 | 0.60 |

Note: ^1^ The premix provides vitamin A 9 500 IU, vitamin D3 500 IU, vitamin E 20 IU, vitamin K 1.2 mg, vitamin B1 2.2 mg, vitamin B2 5.0 mg, vitamin B6 2.0 mg, niacin 30 mg, pantothenic acid 12.0 mg, folic acid 0.8 mg, biotin 0.18 mg, iodine 0.35 mg, selenium 0.30 mg, manganese 100 mg, iron 80 mg, copper 8 mg, zinc 75 mg. The premix does not contain antibiotics or chemically synthesized antimicrobials.

^2^ Metabolizable energy of nutrient components is calculated value, others are measured value.

**Table S2** Content of chemical components in marigold meal

| Items | MM | MF |
| --- | --- | --- |
| Total flavonoid, % | 3.21±0.05 | 4.27±0.15 |
| Total sugars, % | 0.22±0.01 | 0.36±0.01 |
| Total phenol, % | 8.15±0.02 | 8.57±0.02 |
| Total protein, % | 3.29±0.64 | 5.77±0.64 |
| Lutein, g/kg | 0.60±0.06 | 0.58±0.08 |

Note: MM: unfermented marigold meal; MF: fermented marigold meal.

**Table S3** Effects of marigold meal on the growth performance of broilers

| Items | CK | MML | MMH | MFL | MFH | *P*-value |
| --- | --- | --- | --- | --- | --- | --- |
| Initial BW, g | 103.01±2.97 | 100.78±1.53 | 100.03±3.08 | 104.00±3.20 | 103.10±3.55 | 0.857 |
| Final BW, g | 1732.33±39.24 | 1675.33±43.38 | 1667.83±28.48 | 1591.33±50.95 | 1721.30±34.36 | 0.135 |
| ADFI, g | 112.37±2.76^b^ | 116.28±5.28^b^ | 141.86±11.82^a^ | 110.98±1.02^b^ | 109.79±2.44^b^ | 0.005 |
| ADG, g | 38.79±0.94 | 37.48±1.04 | 37.32±0.62 | 35.41±2.81 | 38.52±2.08 | 0.124 |
| F/G | 2.90±0.07^b^ | 3.10±0.12^b^ | 3.81±0.33^a^ | 3.15±0.12^b^ | 2.85±0.03^b^ | 0.005 |

^a-b^ mean difference of different superscripts in the same row, *P*<0.05.

^1^ BW= body weight; ADFI= average daily feed intake; ADG= average daily gain; F/G= feed to weight ratio.

^2^ CK= basal diet (SBM); MML=SBM+5% marigold meal; MMH=SBM+10% marigold meal; MFL=SBM+5% fermented marigold meal; MFH=SBM+10% fermented marigold meal.

^3^ Mean ± SEM (n=6).

**Table S4** Effect of different dosage marigold meal on meat quality of white feather broilers

| Items | CK | MML | MMH | MFL | MFH | *P*-value |
| --- | --- | --- | --- | --- | --- | --- |
| Drip loss (%) | 33.13±2.61^ab^ | 26.42±2.43^b^ | 34.19±2.23^ab^ | 37.34±2.28^ab^ | 42.12±4.25^a^ | 0.012 |
| Cooking loss (%) | 22.82±2.80^b^ | 26.34±1.79^ab^ | 31.57±1.81^a^ | 26.26±2.07^ab^ | 29.14±0.81^ab^ | 0.047 |
| Shear force (N) | 3.01±0.54 | 3.33±0.42 | 3.80±0.39 | 3.02±0.41 | 3.37±0.75 | 0.821 |
| pH45 min | 6.09±0.16 | 6.06±0.02 | 6.09±0.01 | 6.10±0.01 | 6.20±0.03 | 0.733 |
| pH24 h | 5.89±0.04 | 5.86±0.02 | 5.92±0.02 | 5.87±0.04 | 5.82±0.03 | 0.484 |
| L* 45 min | 27.41±0.26 | 27.60±0.27 | 27.47±0.13 | 27.38±0.06 | 26.83±0.29 | 0.184 |
| a* 45 min | 4.55±0.20 | 4.48±0.13 | 4.27±0.08 | 4.54±0.11 | 4.72±0.16 | 0.323 |
| b* 45 min | 9.35±0.17 | 9.54±0.18 | 9.46±0.11 | 9.29±0.13 | 9.16±0.15 | 0.485 |
| L*24 h | 30.81±0.09 | 30.57±0.25 | 30.65±0.22 | 30.20±0.23 | 30.51±0.29 | 0.449 |
| a*24 h | 5.55±0.39 | 5.48±0.30 | 5.74±0.38 | 5.77±0.39 | 5.37±0.28 | 0.920 |
| b*24 h | 9.79±0.15 | 9.81±0.33 | 10.37±0.07 | 10.06±0.19 | 10.02±0.11 | 0.243 |
| Protein g/100g | 23.68±0.52 | 23.95±0.43 | 22.68±0.25 | 23.23±0.40 | 23.08±0.44 | 0.260 |
| Fat g/100g | 1.25±0.06 | 1.35±0.08 | 1.33±0.16 | 1.40±0.10 | 1.43±0.10 | 0.799 |
| Water (%) | 75.19±0.34 | 75.59±0.55 | 74.65±0.34 | 76.62±0.35 | 75.80±0.57 | 0.054 |

^a-b^ Means within a row with different superscript letters are significantly different (*P* < 0.05).

^1^ L*: luminance; a*: redness; b*: yellowness.

^2^ CK= basal diet (SBM); MML=SBM+5% marigold meal; MMH=SBM+10% marigold meal; MFL=SBM+5% fermented marigold meal; MFH=SBM+10% fermented marigold meal.

^3^ Mean ± SEM (n=6).
